# Association of single-nucleotide polymorphisms in the *RAGE* gene and its gene- environment interactions with diabetic nephropathy in Chinese patients with type 2 diabetes

**DOI:** 10.18632/oncotarget.18785

**Published:** 2017-06-28

**Authors:** Ying Zhang, Nan Jia, Feng Hu, Naijun Fan, Xiaohua Guo, Han Du, Changlin Mei, Chunfang Gao

**Affiliations:** ^1^ Institute of Anal-Colorectal Surgery, No. 150th Central Hospital of PLA, Luoyang, 471000, P. R. China; ^2^ Department of Nephrology, Shenzhen Hospital, Southern Medical University, Shenzhen, 518100, P. R. China; ^3^ Department of Nephrology, The Second Affiliated Hospital of the Second Military Medical University, Shanghai, 200003, P. R. China; ^4^ Department of Nephrology, No. 150th Central Hospital of PLA, Luoyang, 471000, P. R. China

**Keywords:** diabetic nephropathy, *RAGE*, single nucleotide polymorphisms, type 2 diabetes, smoking

## Abstract

**Aims:**

To investigate the association of several single nucleotide polymorphisms (SNPs) within *RAGE* gene and additional gene- smoking interaction with diabetic nephropathy (DN) risk in Chinese patients with type 2 diabetes mellitus (T2DM).

**Methods:**

A total of 865 participants (570 males, 295 females) were selected, including 430 T2DM complicated DN patients and 435 controls (T2DM patients without DN). Generalized multifactor dimensionality reduction (GMDR) was used to screen the best interaction combination among SNPs and smoking. Logistic regression was performed to investigate impact of 4 SNPs within *RAGE* gene, additional gene- smoking interaction on DN risk.

**Results:**

DN risk was significantly higher in carriers with the C allele of rs1800625 than those with TT genotype, adjusted OR (95%CI) =1.57 (1.16-2.17), and higher in carriers with the T allele of rs184003 than those with GG genotype, adjusted OR (95%CI) = 1.64 (1.21-2.12). GMDR model indicated a significant two-locus model (p=0.0010) involving rs1800625 and smoking, the cross-validation consistency of this two- locus model was 10/ 10, and the testing accuracy was 60.72%. We also conducted stratified analysis for the significant models in the GMDR analysis by using logistic regression. We found that current smokers with rs1800625- TC or CC genotype have the highest DN risk, compared with never- smokers with rs1800625- TT genotype, OR (95%CI) = 2.92 (1.94 -3.96), after covariates adjustment.

**Conclusions:**

We found that the C allele of rs1800625 and the T allele of rs184003 within *RAGE* gene, interaction between rs1800625 and smoking were all associated with increased DN risk.

## INTRODUCTION

Diabetic nephropathy (DN) was a kind of common diabetes mellitus related microvascular complications and could lead to end-stage renal disease [1]. The determinants of DN are complex, including environmental and genetic factors, which was supported by a strong familial aggregation of DN [2]. The potential biological mechanisms for DN were not well known yet. It is widely accepted that DN is a heterogeneous disorder caused by the interaction between environmental and genetic factors [3]. Increased formation of glucose-derived AGEs one of the major pathways for DN risk. There is growing evidence to suggest that *RAGE* has an important role in diabetic vascular complications [4–6].

Several polymorphisms of the *RAGE* gene had been suggested, and their associations with type 2 diabetes mellitus (T2DM) and T2DM related microvascular complications, such as diabetic retinopathy (DR) or DN had been reported previously [7–9], including (rs1800624 [−374T>A], rs1800625 [−429T>C], rs184003 [1704G>T] and rs2070600 [Gly82Ser]). However, the results were conflicting considering *RAGE* gene polymorphisms with the DN. In addition, DN susceptibility was influenced by not only genetic factors, but also some environment factors, such as smoking [10]. But till now, no study focused on the synergistic effect between *RAGE* gene and smoking on DN risk. So the purpose for this study was to investigate the impact of several SNPs within *RAGE* gene, and their additional interaction with smoking on DN risk in Chinese T2DM patients.

## RESULTS

A total of 865 participants (570 males, 295 females) were selected, including 430 T2DM complicated DN patients and 435 controls (T2DM patients without DN). The mean age was 62.1 ± 13.8 years for all participants. Table 1 shows the general characteristics for cases and controls. The means of age, BMI, HbA1c, FPG and rates for males, alcohol drinking, were not significantly different between cases and controls. The rates for smokers, duration of diabetes more than 6 years, retinopathy and hypertension were higher in cases than controls.

**Table 1:** General characteristics of study participants in case and control group

Variables	Diabetes patients with DN (n=430)	Diabetes patients without DN (n=435)	*P*-values
Age (years)	61.7±15.6	62.4±14.7	0.497
Males N (%)	280 (65.1)	290 (66.7)	0.631
Smoke N (%)	161 (37.4)	126 (31.3)	0.008
Alcohol consumption N (%)	191 (44.4)	178 (40.9)	0.298
Duration of diabetes			
≥6 years	301 (70.0)	226 (52.0)	<0.0001
<6 years	129 (30.0)	209 (48.0)	
Retinopathy N (%)	263 (61.2)	106 (24.4)	<0.0001
BMI (kg/m^2^)	24.1±6.1	23.8±6.3	0.477
FPG (mmol/l)	8.1±2.6	8.3±2.3	0.231
HbA1c (%)	8.51 ± 2.24	8.58 ± 2.26	0.647
Hypertension N (%)	252 (58.6)	135 (31.0)	<0.0001

Note: means± standard deviation for age, FPG, BMI, FPG, fast plasma glucose; BMI, body mass index.

The *P*-values for Hardy–Weinberg equilibrium test in controls were all were more than 0.05. The frequencies for the C allele of rs1800625 and the T allele of rs184003 were significantly higher in T2DM complicated DN patients than controls (30.9%*vs*20.3%, 29.2 *vs*19.5%). DN risk was significantly higher in carriers with the C allele of rs1800625 than those with TT genotype (TC or CC versus TT), adjusted OR (95%CI) =1.57 (1.16-2.17), and higher in carriers with the T allele of rs184003 than those with GG genotype (GT or TT versus GG), adjusted OR (95%CI) = 1.64 (1.21-2.12). However, we found that rs1800624 and rs2070600 were not associated with DN risk after covariates adjustment (Table 2).

**Table 2:** Genotype and allele frequencies of 4 SNPs between case and control group

SNP	Genotypes and Alleles	Frequencies N (%)		OR (95%CI)*	*P*-values	*P*-values for HWE test in controls
		Controls (n=435)	Cases (n=430)			
rs1800624 -374T>A						
	Co-dominant					
	TT	254 (58.4)	224 (52.1)	1.00 (ref)		0.871
	TA	156 (35.9)	168 (39.1)	1.20 (0.78-1.79)	0.521	
	AA	25 (5.7)	38 (8.8)	1.45 (0.73-2.21)	0.607	
	Dominant					
	TT	254 (58.4)	224 (52.1)	1.00 (ref)		
	TA+AA	181 (41.6)	206 (47.9)	1.28 (0.76-1.86)	0.582	
	Allele, A (%)	206 (23.7)	244 (28.4)			
rs1800625 -429T>C						
	Co-dominant					
	TT	280 (64.4)	212 (49.3)	1.00 (ref)		0.237
	TC	133 (30.6)	170 (39.5)	1.33 (1.10-1.77)	0.0002	
	CC	22 (5.0)	48 (11.2)	2.06 (1.42-3.02)	<0.0001	
	Dominant					
	TT	280 (64.4)	212 (49.3)	1.00 (ref)		
	TC+CC	155 (35.6)	218 (50.7)	1.57 (1.16-2.17)	<0.0001	
	Allele, C (%)	177 (20.3)	266 (30.9)			
rs184003 1704G>T						
	Co-dominant					
	GG	285 (65.5)	220 (51.2)	1.00 (ref)		0.301
	GT	130 (29.9)	169 (39.3)	1.43 (1.18-1.81)	<0.0001	
	TT	20 (4.6)	41 (9.5)	2.07 (1.38-2.82)	<0.0001	
	Dominant					
	GG	285 (65.5)	220 (51.2)	1.00 (ref)		
	GT+TT	150 (34.5)	210 (48.8)	1.64 (1.21-2.12)	<0.0001	
	Allele, T (%)	170 (19.5)	251 (29.2)			
rs2070600 Gly82Ser						
	Co-dominant					
	GG	249 (57.2)	216 (50.2)	1.00 (ref)		0.313
	GA	155 (35.6)	172 (40.0)	1.15 (0.75-1.70)	0.457	
	AA	31 (7.1)	42 (9.8)	1.38 (0.70-2.10)	0.618	
	Dominant					
	GG	249 (57.2)	216 (50.2)	1.00 (ref)		
	GA+AA	186 (42.8)	214 (49.8)	1.20 (0.77-1.86)	0.536	
	Allele, A (%)	217 (24.9)	256 (29.8)			

*Adjusted for gender, age, smoking and alcohol status, BMI and WC. Bonferroni correction threshold: p<0.00625.

**Figure 1:**
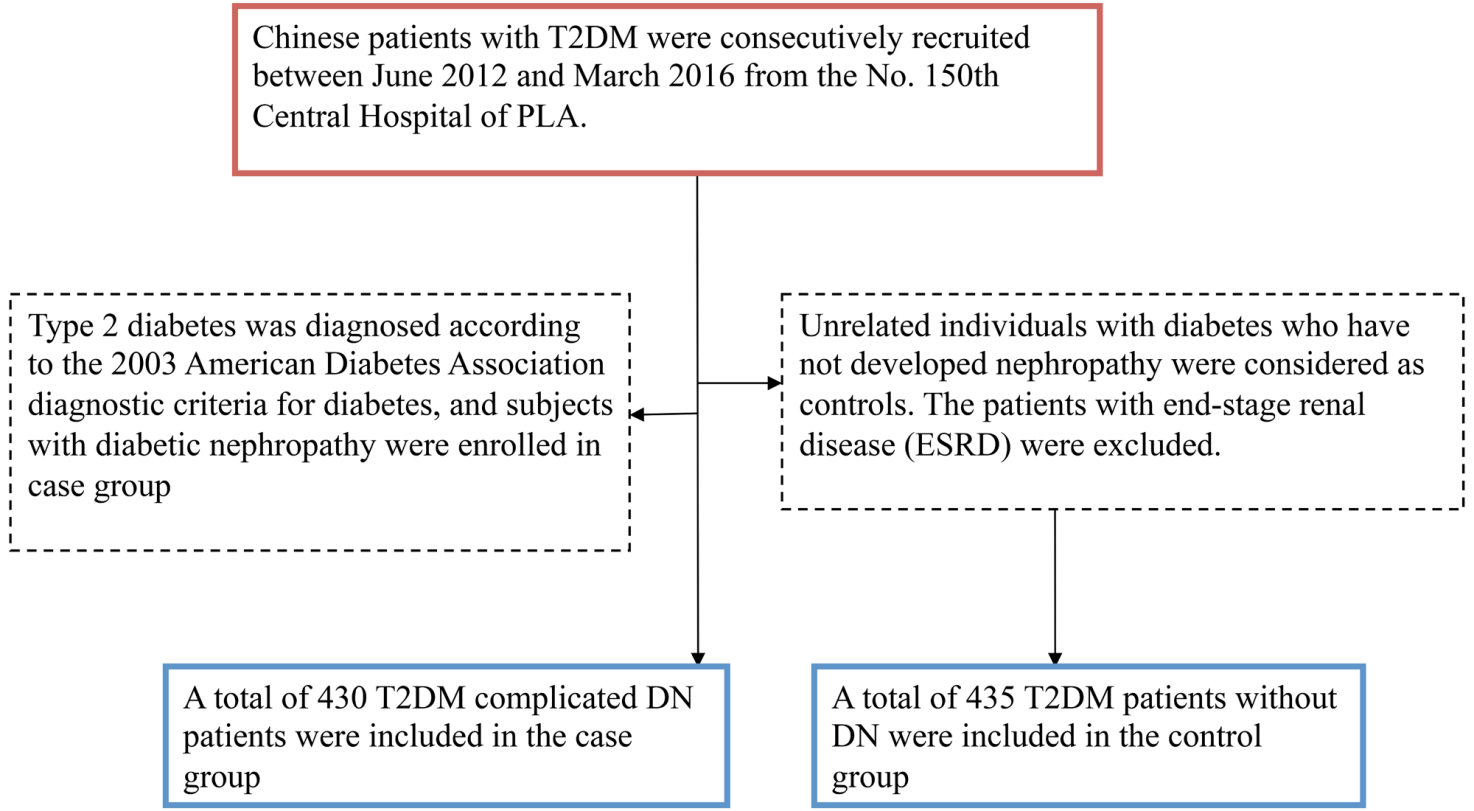
Stratified analysis for rs1800625- smoking interaction using logistic regression

GMDR model were used to screen the best interaction combinations for gene- smoking. We found a significant two-locus model (p=0.0010) involving rs1800625 and smoking (Table 3), the cross-validation consistency of this two- locus model was 10/ 10, and the testing accuracy was 60.72%. We also conducted stratified analysis for the significant models in the GMDR analysis by using logistic regression. We found that current smokers with rs1800625- TC or CC genotype have the highest DN risk, compared with never- smokers with rs1800625- TT genotype, OR (95%CI) = 2.92 (1.94 -3.96), after covariates adjustment. (Figure 1).

**Table 3:** GMDR analysis on the best gene–smoking interaction models

Locus no.	Best combination	Cross-validation consistency	Testing accuracy	*p-values* *
2	rs1800625 Smoking	10/10	0.6072	0.0010
3	rs1800625 rs184003 Smoking	7/10	0.5399	0.1719
4	rs1800625 rs184003 rs1800624 Smoking	6/10	0.5399	0.3770
5	rs1800625 rs184003 rs1800624 rs2070600 Smoking	6/10	0.4958	0.4258

*Adjusted for gender, age, hypertension, duration of diabetes, drinking and BMI

## DISCUSSION

In our study, we found that both the C allele of rs1800625 and the T allele of rs184003 were significantly associated with increased DN risk. However, we found that rs1800624 and rs2070600 were not associated with DN risk after covariates adjustment. Genetic study have identified that approximate 30 polymorphisms occur in the *RAGE* gene [11, 12]. In these identified SNPs, rs1800624 and rs1800625 were two functional and common variants in the *RAGE* gene promoter region [13]. Several studies have reported the relationship between polymorphisms of the *RAGE* gene with T2DM related complications, such as DR and DN [7–9], however, these studies concluded conflicting results. Lindholm et al [14] suggested a significant relationship between the *RAGE* rs1800624 and DN in both type 1 and type 2 DM. Kanková et al [9] also reported *RAGE2* haplotype containing minor alleles at positions 429, 2184 and major allele at position 374 was significantly associated with DN. Another study [15] also shown that polymorphisms in the *AGER* genes increase risk of diabetic micro- and macroangiopathy either alone or together. A recent study [16] suggested that the *RAGE* genes involved in modulation of oxidative pathway could be major contributor to diabetic chronic renal insufficiency. A Chinese study [7] suggested a significant association between *RAGE*-2184A/G polymorphism and DN in Chinese Han patients with T2DM. A meta- analysis [17] consist of 8 studies, enrolled a total of 1725 cases and 1857 controls indicated no association between *RAGE* gene and DN. However, in the recessive model, this study showed a marginal association, and they concluded that the *RAGE* gene -429CC genotype might be a risk factor for DN in patients with T2DM.

DN susceptibility was influenced by both genetic and environment factors, and previously several environmental factors associated with DN were reported, and in these risk factors, cigarette smoking, which was a new and modifiable factor, has been suggested to play a crucial role in increasing the risk of DN risk [10, 18, 19]. In current study, the rate of smoking was higher in DN cases than controls, so in this study we also investigated the association between *RAGE* gene- smoking interaction and DN risk. GMDR model were used to screen the best interaction combinations for gene- smoking interaction. We found a significant interaction involving rs1800625 and smoking, current smokers with rs1800625- TC or CC genotype have the highest DN risk, compared with never- smokers with rs1800625- TT genotype. The results of this study suggested that the risk of DN may be modified by some lifestyle factors, such as smoking. The genetic variant (rs1800625) within *RAGE* gene interact with smoking could influence susceptibility to DN.

There several limitations in our study. Firstly, data on the serum levels of sRAGE were not measured and, therefore, we could not investigate whether the serum levels of sRAGE were concomitantly associated with these four SNPs of the *RAGE*. Secondly, more environmental risk factors should be investigated in the future studies. Thirdly, the results obtained from this study should be checked in studies with larger sample size.

In conclusion, we found that the C allele of rs1800625 and the T allele of rs184003 within *RAGE* gene, interaction between rs1800625 and smoking were all associated with increased DN risk.

## MATERIALS AND METHODS

### Subjects

**Figure 2:**
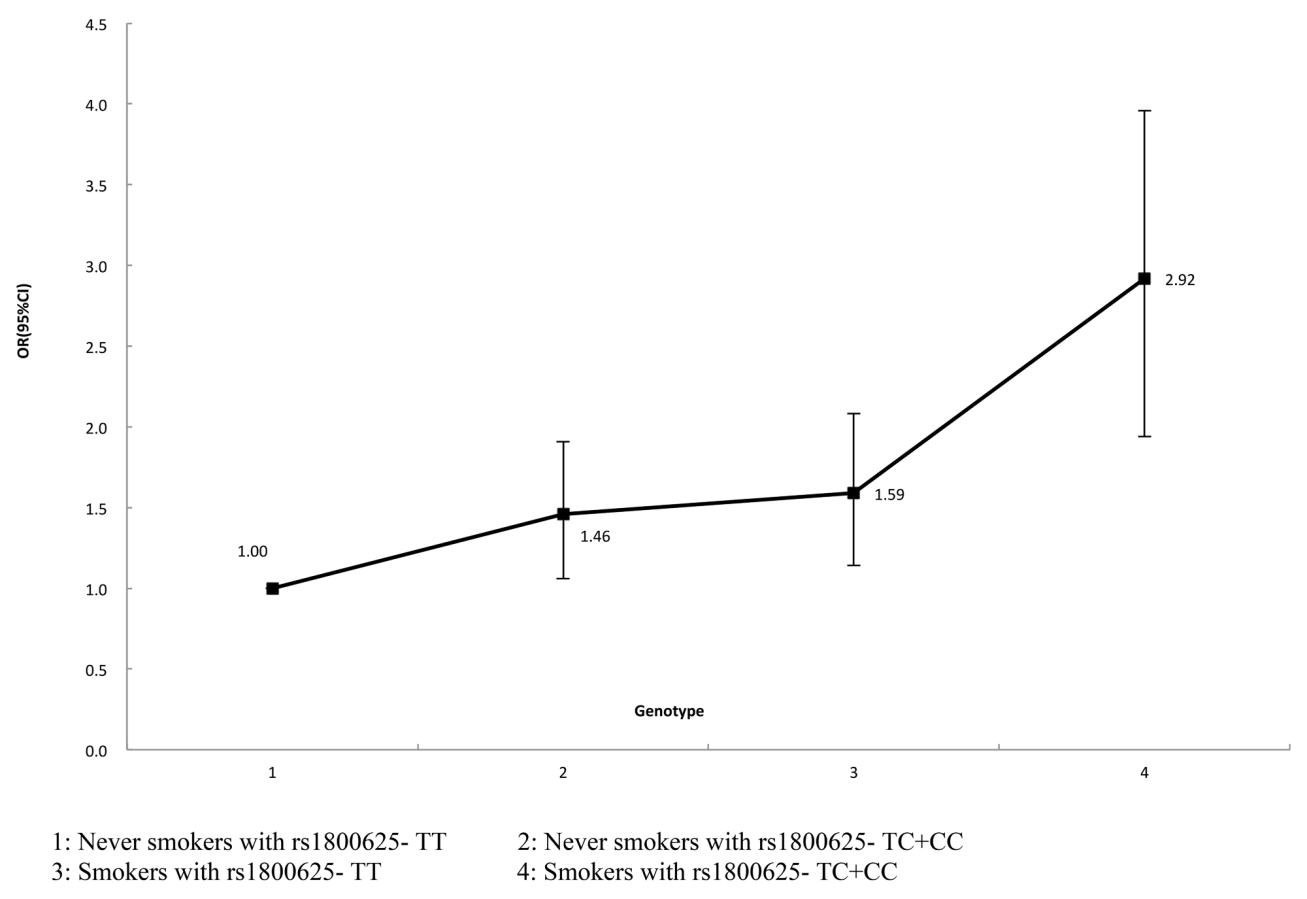
A flowchart on study population selection and exclusion

Chinese patients with T2DM were consecutively recruited between June 2012 and March 2016 from the No. 150th Central Hospital of PLA. Type 2 diabetes was diagnosed according to the 2003 American Diabetes Association diagnostic criteria for diabetes, and subjects were divided into two groups: without diabetic nephropathy (n = 435) and with diabetic nephropathy (n = 430) according to their 24-hour albumin excretion rate (AER) and estimated glomerular filtration rate (eGFR). Individuals with T2DM and nephropathy were considered as cases, and unrelated individuals with diabetes who have not developed nephropathy were considered as controls. The patients with end-stage renal disease (ESRD) were excluded (Figure 2). All demographic and related clinical data including residential region, age, ethnicity, and education status were collected through a face-to-face questionnaire and a review of medical records. All participants underwent detailed clinical evaluation, and followed by biochemical investigations. Blood samples were collected from each participant in the morning after at least 8 hours of fasting. HbA1c content was measured using a Bio-Rad D-10 glycated hemoglobin analyzer (Bio-Rad, Hercules, USA). Informed consent was obtained from all participants.

### Genomic DNA extraction and genotyping

The SNPs were selected based on the NCBI database (http://www.ncbi.nlm.nih.gov/projects/SNP). Taking into account the limited human resources and financial resources, just 4 SNPs within *RAGE* gene were selected for genotyping, including: rs1800625, rs184003, rs1800624 and rs2070600. Genomic DNA from participants was extracted from EDTA-treated whole blood, using the DNA Blood Mini Kit (Qiagen, Hilden, Germany) according to the manufacturer’s instructions. Genotyping was performed using a thermocycler PCR system, followed by a restriction fragment length polymorphism (RFLP) assay. For each SNP, the PCR was conducted in a reaction volume of 25 μl, consisting of 1 μl of each specific primer, 2 μl of genomic DNA, 12.5 μl of Green PCR Master Mix (Shanghai Sangon Biotech Co., Ltd., China), and 8.5 μl of nuclease-free water. The nucleotide sequence of primers and description for the 4 SNPs were shown in Table 4. The PCR conditions for these four SNPs were all as follows: initial denaturation at 95 °C for 5 min, 30 cycles of denaturation at 95 °C for 30 s, annealing at 61 °C for 30 s, extension at 72 °C for 45 s, and a final extension at 72 °C for 10 min.

**Table 4:** Description and primer sequence for 4 SNPs used for PCR analysis

SNP ID	Chromosome	Functional Consequence	Restriction enzymatic	Major/minor alleles	Primer sequences
rs1800624 -374T>A	6:32184610	downstream variant 500B, nc transcript variant, upstream variant 2KB, utr variant 5 prime	*Mun*I	T/A	F: 5’-GGGCAGTTCTCTCCTCACTT-3’ R: 5’-CGTCTTGTCACAGGGAATGC-3’
rs1800625 -429T>C	6:32184665	downstream variant 500B, nc transcript variant, upstream variant 2KB, utr variant 5 prime	AluI	C/T	F: 5’-GGGCAGTTCTCTCCTCACTT-3’ R: 5’-CGTCTTGTCACAGGGAATGC-3’
rs184003 1704G>T	6:32182519	Intron variant	*FspB*I	G/T	F: 5’-GAGACAGGGCTCTTCACACT-3’ R: 5’-TTTCCCTCGTTAGCCCTCTG-3’
rs2070600 Gly82Ser	6:32183666	Missense, nc transcript variant	AluI	G/A	F: 5’-GAAGGTCCTGTCTCCCCAG-3’ R:5’-GTAAGAGGGAGGCCTTGGAG-3’

### Statistical analysis

All statistical analyses were performed using the SPSS 22.0 software package (SPSS Inc, Chicago) for Windows 7. Categorical variables were presented as absolute values and percentages, and continuous variables were expressed as means ± standard deviations (SD). Student’s t test was used to compare continuous variables, while Chi-square test was used to compare categorical variables between cases and controls. Hardy-Weinberg equilibrium (HWE) examination was used by SNPstats (http://bioinfo.iconcologia.net/SNPstats). Generalized multifactor dimensionality reduction (GMDR) model was used to analyze the gene- smoking interaction, some parameters including cross-validation consistency, the testing balanced accuracy and the sign test were calculated, a sign test or a permutation test (providing empirical *p*-values) for prediction accuracy can be used to measure the significance of an identified model. Logistic regression was performed to investigate association between 4 SNPs within *RAGE* gene and DN risk, and used for stratified analysis on significant interaction combination obtained from GMDR. All reported p-values were two-tailed, and to correct for multiple testing we defined a Bonferroni corrected- threshold in different tables.
